# Modulation of the Functional State of Mouse Neutrophils by Selenium Nanoparticles In Vivo

**DOI:** 10.3390/ijms232113651

**Published:** 2022-11-07

**Authors:** Valentina N. Mal’tseva, Sergey V. Gudkov, Egor A. Turovsky

**Affiliations:** 1 Institute of Cell Biophysics of the Russian Academy of Sciences, Federal Research Center “Pushchino Scientific Center for Biological Research of the Russian Academy of Sciences”, 142290 Pushchino, Russia; 2 Prokhorov General Physics Institute of the Russian Academy of Sciences, 38 Vavilove St., 119991 Moscow, Russia

**Keywords:** neutrophils, selenium nanoparticles, ROS production, adhesion, NETs, genes, RT-PCR

## Abstract

This study aimed to discover the immunomodulatory effect of selenium nanoparticles (SeNPs) on the functional state of neutrophils in vivo. Intraperitoneal injections of SeNPs (size 100 nm) 2.5 mg/kg/daily to BALB/c mice for a duration of 7–28 days led to the development of an inflammatory reaction, which was registered by a significant increase in the number of neutrophils released from the peritoneal cavity, as well as their activated state, without additional effects. At the same time, subcutaneous injections of the same SeNPs preparations at concentrations of 0.1, 0.5, and 2.5 mg/kg, on the contrary, modulated the functional state of neutrophils depending on the concentration and duration of SeNPs administration. With the use of fluorescence spectroscopy, chemiluminescence, biochemical methods, and PCR analysis, it was found that subcutaneous administration of SeNPs (0.1, 0.5, and 2.5 mg/kg) to mice for a short period of time (7–14 days) leads to modification of important neutrophil functions (adhesion, the number of migrating cells into the peritoneal cell cavity, ROS production, and NET formation). The obtained results indicated the immunostimulatory and antioxidant effects of SeNPs in vivo during short-term administration, while the most pronounced immunomodulatory effects of SeNPs were observed with the introduction of a low concentration of SeNPs (0.1 mg/kg). Increase in the administration time of SeNPs (0.1 mg/kg or 2.5 mg/kg) up to 28 days led to a decrease in the adhesive abilities of neutrophils and suppression of the expression of mRNA of adhesive molecules, as well as proteins involved in the generation of ROS, with the exception of NOX2; there was a tendency to suppress gene expression pro-inflammatory factors, which indicates the possible manifestation of immunosuppressive and anti-inflammatory effects of SeNPs during their long-term administration. Changes in the expression of selenoproteins also had features depending on the concentration and duration of the administered SeNPs. Selenoprotein P, selenoprotein M, selenoprotein S, selenoprotein K, and selenoprotein T were the most sensitive to the introduction of SeNPs into the mouse organism, which indicates their participation in maintaining the functional status of neutrophils, and possibly mediated the immunomodulatory effect of SeNPs.

## 1. Introduction

Neutrophils are the main effector cells in the innate immune system. They provide the first line of defense against invading pathogens and their own defective cells (tumor- and viral-transformed cells) and are involved in the regulation of the inflammatory processes that accompany many diseases [1,2]. Neutrophils have a wide arsenal of antimicrobial mechanisms—migration to the area of damage and inflammation, phagocytosis (oxygen-dependent and oxygen-independent mechanisms), generation of reactive oxygen species (ROS), release of extracellular traps of neutrophils (NET), and production of numerous spectra of mediators, including inflammatory factors (cytokines) [2,3]. Nowadays, research on neutrophils is in its enhancement; new roles of neutrophils are being discovered not only in the mechanisms of innate immunity, but also in adaptive immunity, as well as homeostasis maintenance at the level of the whole organism. This is especially significant in diseases associated with chronic inflammation, oncogenesis, angiogenesis, autoimmunity, and wound healing [2,4,5,6]. Netosis is an example of a relatively new function of neutrophils. This is a novel killing mechanism by which neutrophils release neutrophil extracellular traps (NETs) containing nuclear DNA coated with the histones, proteases, and granular and cytosolic proteins necessary for the capture and elimination of a variety of pathogens, including bacteria, parasites, and fungi [7,8,9,10].

In addition to the undeniable “positive” role of the neutrophil in the body, researchers have identified a range of pathological conditions, including autoimmunity and cancer, when neutrophil activity can be harmful to the body [11,12]. Thus, uncontrolled cytotoxic activity, excessive formation of ROS, and NETs can initiate tissue damage that can cause chronic inflammation and autoimmune disease [11,13,14].

Selenium (Se) has been shown to be an important micronutrient; its level affects the functioning of the immune system [15,16,17]. Adequate Se intake has been shown to play an important role in the functions of various innate immune cells, including granulocytes, monocytes and macrophages, dendritic cells, and natural killer cells [18,19,20,21,22]. Thus, insufficient intake of Se or other factors (for example, defects in selenoprotein gene expression or some chronic infections that deplete it) can impair innate immunity [15,23] and lead to immunosuppression, whereas the addition of Se leads to the restoration or enhancement of immunological functions [24]. It is suggested that the immunostimulatory activity of Se is largely related to free radical elimination, ROS neutralization, and reduction of oxidative stress [25].

The biological effect of Se mainly depends on the inclusion of this metalloid in selenoproteins in the form of the amino acid selenocysteine, which is located in the active center of proteins. Immune cells are not significantly different from most cell types in terms of selenoprotein expression patterns. They are characterized by a higher level of expression of selenoproteins, which regulate redox processes and the process of protein folding [16,24,26]. There have only been a few research papers on the specific role of selenoproteins in the functioning of neutrophils. These studies have an episodic character and are usually associated with the investigation of additional consumption of Se in animal farming in order to enhance animal resistance to diseases [27,28,29].

Of particular interest as a source of Se are nanoparticles (SeNPs). SeNPs are a unique form of Se that is characterized by being a low-dispersive bioactive form with powerful antioxidant abilities. The distinguishing characteristics of SeNPs are: large surface area, high surface activity, strong adsorption capacity, chemical stability, high bioavailability, and minimal toxic effects on humans and animals [30,31,32]. Due to their small size (10–100 nm), SeNPs are not absorbed by the reticuloendothelial system, but are large enough not to be filtered out in the kidneys [33]. The potential therapeutic benefits of SeNPs have been shown in various diseases mediated by oxidative stress and inflammation, such as arthritis, cancer, diabetes, nephropathy, and ischemia [34,35,36]. In addition, SeNPs can be used as drug delivery vehicles or biomacromolecules for chemotherapy [37,38]. The effect of SeNPs on tissues and cells depends on their characteristics, including size, shape, dose, concentration, type of nanoparticles, and exposure period [39,40]. For example, SeNPs 5–200 nm in size actively inactivate free radicals, while particles 5–15 nm in size are less effective but prevent DNA oxidation. At the same time, SeNPs are most biologically effective in the range of concentrations below 0.5 mM, whereas Na_2_SeO_3_ has an IC50 > 2.5 mM [26].

SeNPs have been shown to play a central role in regulating the immune response to various bacterial and viral antigens by activating immune system cells, macrophages, and T effector cells [41,42]. As a rule, the action of SeNPs has been investigated based solely on their antioxidant properties and applied in pathologies. For example, the addition of 0.2 mg/kg SeNPs per day led to a decrease in the number of immune system cells and a decrease in the inflammatory response in rats with spinal cord injury [43]. Recent studies have shown that SeNPs as an adjuvant can induce protective immune responses in vaccines against bacteria and viruses [44,45]. Interestingly, SeNPs are able to target macrophages and regulate their polarization, initiating innate immunity to antimicrobial action and regulating cytokine production [46]. SeNPs may also act as immunomodulatory agents to inhibit tumor growth by enhancing antitumor immune responses involving tumor-associated macrophages and activation of specific T cells [47]. SeNPs may also be candidates for further evaluation as agents for the prevention of immunosuppression under chemotherapy. Both radiation therapy and chemotherapy, which are currently used to treat cancer, are myelosuppressive, initiating a significant suppression of the immune system, and cause serious side effects in cancer patients, increasing the risk of infectious diseases caused by opportunistic microorganisms. These superinfections can significantly worsen the condition of cancer patients. In vivo tests in cytoxan-induced immunosuppressed mice showed that daily supplementation of SeNPs/β-glucan at concentrations of 6 mg/kg significantly stimulated the production of cellular immune factors (leukocytes, neutrophils, lymphocytes, B cells, CD4+ cells, CD34+ cells, and natural killers) [48]. Taken together, all of these works suggest that SeNPs can serve as a new immunomodulator against various bacterial infections, cancer, and other diseases associated with immune system dysfunction, thus, opening up new therapeutic possibilities.

Research of the immunomodulating effects of SeNPs has been widely presented. However, the mechanisms of the modifying effect on the functional state of neutrophils and the effective amounts of SeNPs, as well as their effect on selenoproteins and their participation in immunomodulation, remain uninvestigated. The aim of this study was to investigate the dynamics of the immunomodulatory effect of different concentrations of SeNPs on the functional activity of neutrophils depending on the time of administration to mice.

## 2. Results

### 2.1. Effect of SeNP Injections into Mice on Neutrophil Counts and Their Adhesive Functions

**Figure 1:**
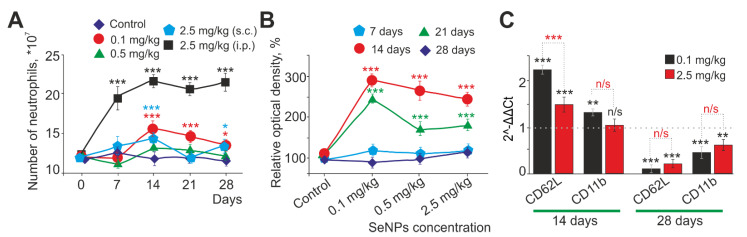
Effect of injection of various concentrations of SeNPs into mice on the neutrophil number and adhesion. (**А**)—Dose- and time-dependent increase in the number of neutrophils isolated from mice peritoneal cavities after zymosan injection. The curves show the counts of neutrophil migration induced by zymosan (5 mg/mL) at 5 h after the daily intraperitoneal injection of SeNPs or saline (control) on days 7, 14, 21, and 28. Abbreviations: 2.5 mg/kg (s.c.)—subcutaneous injections of SeNPs and 2.5 mg/kg (i.p.) intraperitoneal injections of SeNPs. The data are the mean values ± SEM of seven mice. * *p* < 0.01 compared with respective control group. Peritoneal evoked neutrophils of male mice of outbreed strain BALB/c were used in the experiments. Neutrophils comprised nearly 98% of the total number of cells, as determined by acridine orange staining. (**B**)—Dose- and time-dependent modification of neutrophil adhesion on days 7, 14, 21, and 28 after daily injection of SeNPs or saline (control). Concentration-dependent effects of SeNPs (0.1 mg Se/kg, 0.5 mg Se/kg, 2.5 mg Se/kg) on mouse neutrophil adhesion: сurves shown are blue—on day 7, red—on day 14, green—on day 21, and dark blue—on day 28 of administration of SeNPs, respectively. Adhesion was determined by spectrophotometric analysis at 492 nm and then OD492 in the neutrophils from experimental groups with administration of SeNPs normalized to the same parameter measured in control group. Data presented are the mean ± SEM (*n* = 7). Statistical analyses of experimental groups vs. control were performed with paired *t*-test. Significance between group means: * *p* < 0.05, ** *p* < 0.01, and *** *p* < 0.001. Values not marked with an asterisk are invalid. (**C**)—Effect of SeNP injection in vivo on expression of neutrophil adhesion molecules CD11b and CD62L. Relative mRNA expression of genes encoding of CD11b and CD62L in neutrophils on days 14 and 28 after daily injection of 0.1 mg Se/kg (black bars) and 2.5 mg Se/kg (red bars) SeNPs. Dashed line—level of gene expression in controls (mice with administration of saline). GAPDH was used as housekeeping gene. Data are shown as mean ± SEM of five independent experiments. Statistical analysis of experimental groups versus control was performed with paired *t*-test and is marked with black asterisks. Significance between experimental groups is marked with red asterisks: *** *p* < 0.001 and ** *p* < 0.01; n/s—no significance.

Neutrophils were isolated from the peritoneal cavity of mice after induction of inflammation with a single intraperitoneal injection of a suspension of zymosan (5 mg/mL, i.p.). It turned out that, in the experimental group of mice, which were injected intraperitoneally with 2.5 mg/kg SeNPs for 28 days, there was a significant increase in the number of peritoneally evoked neutrophils on the 7th, 14th, 21st, and 28th days compared with the subcutaneously injected experimental groups (Figure 1A, black curve). Subcutaneous administration of 2.5 mg/kg SeNPs caused a significantly less pronounced increase in the number of neutrophils and only on days 7 and 14 after the injections (Figure 1A, blue curve) and then the number of neutrophils did not differ from the control group of mice. Subcutaneous administration of 0.1 mg/kg SeNPs also resulted in a significant increase in the number of neutrophils after 14 days of administration and beyond (Figure 1A, red curve) compared with the control. Subcutaneous administration of 0.5 mg/kg SeNPs did not cause significant changes in the number of neutrophils (Figure 1A, green curve).

The functional activity of neutrophils can be assessed by their ability to adhere. It was found that, for all experimental groups which were injected subcutaneously with 0.1, 0.5, and 2.5 mg/kg of SeNPs, a similar change in the adhesive properties of neutrophils was characteristic; on the 7th day, they differed slightly from the control, then the adhesive properties more than doubled on the 14th day, then decreased (on the 21st day) until the 28th day, when they returned almost to the control values (Figure 1B). In mice treated with 0.1 mg/kg SeNPs, the adhesive property dynamics had their own characteristics; there was a maximum increase in adhesion among the experimental groups on the 14th day after SeNP administration. After that, there was a gradual decrease in the adhesive properties to control values (Figure 1B).

Based on the obtained data, in order to analyze the level of expression of the genes of the main adhesion molecules of neutrophils—CD62L (L-selectin) and CD11b (β2-integrin)—we chose the critical points of the possible functional changes in neutrophils. These were day 14, when the maximum changes in parameters were observed in all experimental groups, and day 28, when there was a tendency of the parameters to return to control values. PCR analysis was performed in two experimental groups of mice which received subcutaneous injections of SeNPs with low (0.1 mg/kg) and high concentrations (2.5 mg/kg). Both high (2.5 mg/kg) and low (0.1 mg/kg) concentrations of injected SeNPs for 14 days led to a significant increase in the expression of genes encoding the adhesive molecules CD62L and CD11b. On the contrary, on day 28, we observed a significant decrease in expression (Figure 1C). The greatest increase in expression was observed for CD62L in the experimental group with 0.1 mg/kg SeNPs (by 2.3 times) on day 14 and a significant suppression (by 88%) on day 28 (Figure 1C).

Therefore, SeNPs (0.1, 0.5, and 2.5 mg/kg) administered subcutaneously exhibited immunostimulatory properties, causing an increase in the adhesive abilities of neutrophils. As a result, it caused an increase in the number of cells migrating into the peritoneal cavity, which was accompanied by an increase in the expression of adhesive molecules on the 14th day. With prolonged administration of SeNPs for 28 days, the described parameters normalized.

### 2.2. Dose-Dependent Effect of SeNP Injections on Spontaneous and Activated ROS Production by Mouse Neutrophils

**Figure 2:**
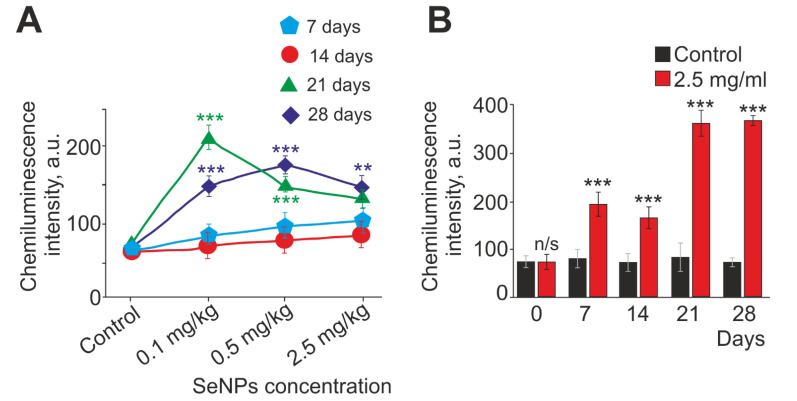
Effect of SeNP injections in vivo on spontaneous ROS production by neutrophils. (**А**)—The changes in spontaneous ROS production in neutrophils of the control and experimental groups with injection of 0.1 mg Se/kg, 0.5 mg Se/kg, and 2.5 mg Se/kg SeNPs. Data are presented at 7 (blue curve), 14 (red), 21 (green), and 28 days (dark blue) after daily injection. Control is the control group with injection of saline. (**В**)—Dynamics of changes in spontaneous ROS production in neutrophils of the control (red) and experimental group with intraperitoneal injection of 2.5 mg Se/kg (black) were estimated at 0, 7, 14, 21, and 28 days after daily injection of SeNPs or saline (control). The neutrophil ROS generation was determined by luminol-dependent chemiluminescence with automated multiplate reader (Spark™ 10M multimode microplate reader). Data are shown as the mean of chemiluminescence intensity, arb.units ± SEM of five animals for each group. Statistical analysis of experimental groups versus control was performed with paired *t*-test. Significance between group means: *** *p* < 0.001 and ** *p* < 0.01. Values not marked with an asterisk are n/s—no significance.

**Figure 3:**
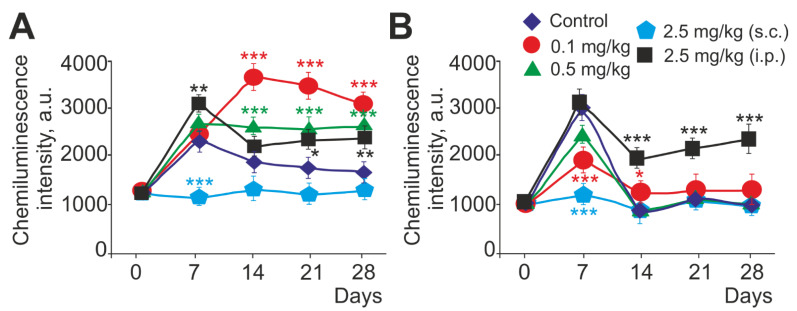
Effect of injection of various concentrations of SeNPs for 28 days on activated ROS production by neutrophils. (**A**,**B**)—Dynamics of changes in ROS production in response to 1 μM FMLF (**A**) and 1 mg/mL OZ (**B**) in neutrophils of control mice and mice of experimental groups with injection of SeNPs: 0.1 mg Se/kg (red), 0.5 mg Se/kg (green), 2.5 mg Se/kg (blue), s.c. (subcutaneous), and 2.5 mg Se/kg (black), i.p. (intraperitoneally). The control group (saline)—dark-blue curve. The neutrophil ROS generation was determined by luminol-dependent chemiluminescence with automated multiplate reader (Spark™ 10M multimode microplate reader). Total ROS production was calculated as the area under the curve of chemiluminescence intensity in time. Data are expressed as the mean chemiluminescence intensity arb.units ± SEM of six animals for each group. Statistical analysis of experimental groups versus control was performed with paired *t*-test. Significance between group means: *** *p* < 0.001, ** *p*< 0.01, and * *p* < 0.05.

Neutrophils are specialized cells that generate active forms (ROS) due to the enzymatic complex of NADPH oxidase. Neutrophil NADPH oxidase is able to produce ROS both spontaneously and through the activation of certain receptors. We evaluated the spontaneous production of ROS by neutrophils (Figure 2), as well as the production of ROS upon activation of neutrophils with either 1 μM FMLF or 1 mg/mL of opsonized zymosan (OZ) (Figure 3). According to our results, the level of spontaneous ROS production was significantly increased (by more than five times until day 28) in animal neutrophils with intraperitoneal injection of 2.5 mg/kg SeNPs, whereas spontaneous ROS production remained practically unchanged in the control (Figure 2A,B). In the experimental groups with subcutaneous injection of SeNPs, this parameter increased in the groups of 0.1 mg/kg, 0.5 mg/kg, and 2.5 mg/kg SeNPs on days 7 and 14 but remained above the control values. On days 21 and 28, a significant increase in the level of spontaneous ROS production was observed at all concentrations of SeNPs (Figure 2A,B). It is essential to highlight the group with subcutaneous injection of 0.1 mg/kg SeNPs. Here, the reversible influence of SeNPs on the spontaneous production of ROS was observed; during long periods of administration of SeNPs (21 and 28 days), there was a significant increase in the level of ROS spontaneous production. A particularly large increase (by almost three times) in this parameter was observed on day 21 (Figure 2A,B).

Neutrophils realize their functional activity by migrating to the focus of inflammation along the gradient of chemotactic factors. The most powerful activators of neutrophil functions, including respiratory burst, are the chemotactic peptide N-formyl-Met-Leu-Phen (FMLF) and opsonized zymosan (OZ).

When assessing changes in the intensity of the respiratory burst activated by 1 μM FMLF, similar dynamics of changes were observed in the experimental groups with subcutaneous injection of 0.5 and 2.5 mg/kg SeNPs and intraperitoneal injection of 2.5 mg/kg SeNPs. On the seventh day, there was an increase then a slight decrease (14–28 days) in activated ROS production, but the level of ROS production remained significantly higher than the control level (Figure 3A). The nature of the changes in the respiratory burst activated by 1 μM FMLF in the group with subcutaneous injection of 0.1 mg/kg SeNPs had characteristic features; the maximum production of ROS was shifted in time, and its significant increase (more than 2.7 times) was observed on the 14th day and not on the 7th, as in other experimental groups. Then, there was a gradual decrease in the intensity of the activated respiratory burst, which, nevertheless, remained significantly higher than in the control on the 28th day (Figure 3A).

A general trend in ROS production on the 14th, 21st, and 28th days was observed upon activation of the respiratory burst with 1 mg/mL OZ. With an increase in the concentration of SeNPs administered subcutaneously, a slight decrease in the studied parameter occurred. The exception was day 7, when, in all experimental groups with subcutaneous injection of SeNPs, there was an increase in ROS production activated by 1 mg/mL OZ (Figure 3C).

Therefore, the results show that the administration of SeNPs for 28 days significantly enhanced the production of ROS activated by 1 μM FMLF compared to in the control group, with the highest activity of the respiratory burst occurring on the seventh day. The exception was the group with an injection of 0.1 mg/kg SeNPs, where the maximum production of ROS was registered on the 14th day. It was noted that ROS level activated by 1 μM FMLF increased inversely with the dose of SeNPs administered. When the respiratory burst was activated with 1 mg/mL OZ, maximum ROS production was also observed on the seventh day in all experimental groups, then, in contrast to the activation of FMLF, there was a decrease in ROS production to approximately control values. This may indicate a general immunostimulatory effect of SeNPs on neutrophils at non-chronic periods of administration (7–14 days) regardless of the nature of the ROS-activating stimulus.

The ROS production level by neutrophils is mediated by their oxidative status, that is, the balance of systems responsible for the production and elimination of ROS. Next, the change in the level of expression of genes encoding proteins of the cell redox status was assessed. PCR analysis was performed in two experimental groups—mice that received daily SeNPs at concentrations of 0.1 and 2.5 mg/kg subcutaneously for 14 and 28 days.

**Figure 4:**
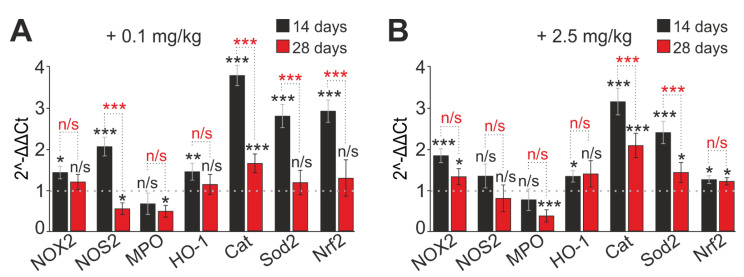
Changes in intracellular expression of neutrophil genes involved in redox homeostasis after administration of 0.1 (**A**) and 2.5 (**B**) mg/kg SeNPs for 14 and 28 days. Neutrophils prepared from mice 14 (black bars) and 28 (red bars) days after daily injection of 0.1 mg Se/kg (**A**) and 2.5 mg Se/kg (**B**) SeNPs. GAPDH was used as housekeeping gene. Dashed line—level of gene expression in controls (mice with administration of saline). Data are expressed as mean ± SEM of five independent experiments. Statistical analysis of experimental groups versus control was performed with paired *t*-test and marked with black asterisks. Significance between experimental groups marked with red asterisks: *** *p* < 0.001, ** *p* < 0.01, and * *p* < 0.05; n/s—no significance.

Both high (2.5 mg/kg) and low (0.1 mg/kg) concentrations of SeNPs led to a significant increase in the expression of genes encoding antioxidant proteins: HO-1, SOD2, Nrf2, and catalase. On the contrary, the expression of genes encoding ROS-producing enzymes NOS2 and MPO was suppressed on day 28, whereas NOS2 expression increased on day 14 (Figure 4A,B). The expression of the NOX2 gene neutrophilic NADPH oxidase, the main ROS generator in neutrophils, was significantly increased in both experimental groups at all periods of SeNP administration, but, with an increase in the duration of SeNP administration (up to 28 days), its expression significantly decreased (Figure 4A,B). It was noted that Nrf2 gene expression increased in the group with 0.1 mg/kg injected SeNPs (by 2.9 times), and the effects of enhancing the expression of antioxidant genes were more pronounced than for a concentration of 2.5 mg/kg SeNPs (Figure 4A).

Therefore, SeNP administration to mice at concentrations of 0.1 and 2.5 mg/kg leads to an increase in spontaneous and stimulated ROS production over short periods. Neutrophil function modulation under the action of SeNPs is accompanied by a change in the expression of mRNA of proteins, providing these functions; there is a tendency to suppress the genes of proteins involved in the ROS generation (with the exception of NOX2) and a significant increase in the expression of antioxidant protein genes. All this shows the immunostimulatory (or priming) and antioxidant properties of SeNPs towards neutrophils, and the maximum effects were manifested at a low concentration of SeNPs (0.1 mg/kg).

### 2.3. The Action of SeNP Injections into Mice Regulates the Inflammatory Status of Neutrophils

**Figure 5:**
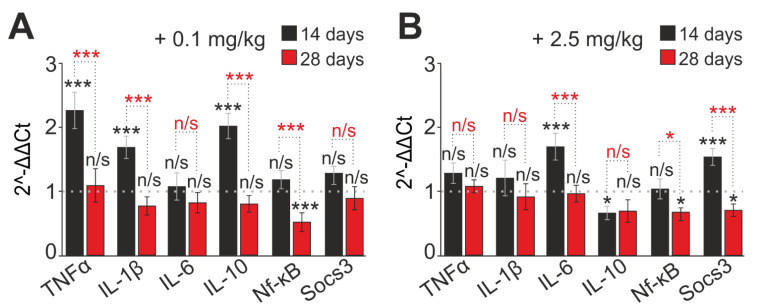
The introduction of 0.1 (**A**) and 2.5 (**B**) mg/kg SeNPs for 14 and 28 days impacted the expression of genes associated with inflammation. Neutrophils prepared from mice 14 (black bars) and 28 (red bars) days after 0.1 mg Se/kg (**A**) and 2.5 mg Se/kg (**B**) SeNPs were injected daily. GAPDH was used as housekeeping gene. Data were analyzed by 2^(-ΔΔCT)^ method. Dashed line—level of gene expression in controls (mice with administration of saline). Data are expressed as mean ± SEM of five independent experiments. Statistical analysis of experimental groups versus control was performed with paired *t*-test and marked with black asterisks. Significance between experimental groups marked with red asterisks: *** *p* < 0.001 and * *p* < 0.05; n/s—no significance.

The functional state of neutrophils upon administration of SeNPs to mice, in addition to the Red/Ox status, can also be characterized by changes in the inflammatory status of cells. Administration of 0.1 and 2.5 mg/kg SeNPs to mice for 14 days resulted in a significant increase in the expression of mRNA of inflammatory factors TNFa, IL1b, IL6, NF-kB, and SOCS3 (Figure 5A,B), whereas a longer application of the used concentrations of SeNPs (28 days) led to a decrease in the expression of the listed genes (Figure 5). The SeNP concentration injected into the experimental animals impacted the expression of the anti-inflammatory cytokine IL10. For instance, at a low concentration of SeNPs (0.1 mg/kg), expression growth was observed (Figure 5A). On the contrary, at a high concentration of SeNPs (2.5 mg/kg)m suppression was observed (Figure 5B). On the 28th day of SeNP administration, IL10 expression was inhibited in both experimental groups (Figure 5A,B).

The immunomodulatory effect of SeNPs was observed when assessing the inflammatory status of neutrophils. For a short term (14 days), there was an increase in the expression of mRNA of inflammatory factors, which may indicate the immunostimulatory effects of SeNPs. For chronic administration (28 days), there was a decrease in the expression of the studied inflammatory factors, which may indicate the immunosuppressive and anti-inflammatory effects of SeNPs.

### 2.4. Dose-Dependent Effect of SeNPs on the Antimicrobial Activity of Mouse Neutrophils Mediated by NET Generation

**Figure 6:**
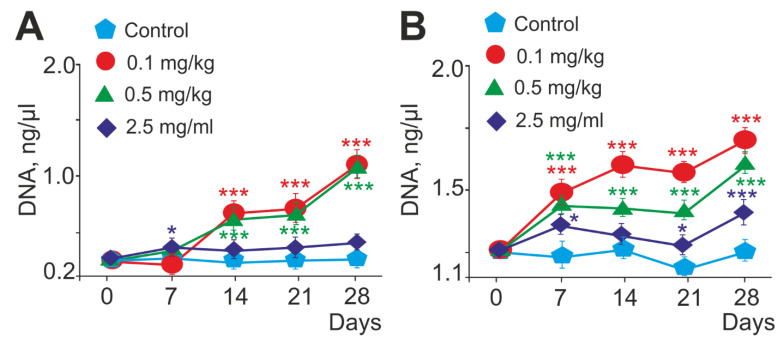
SeNPs modified unstimulated and stimulated NETs formation in a dose-dependent manner. NETs were released by unstimulated (neutrophils alone) (**A**) and stimulated (neutrophils + 1 mg/mL zymosan) (**B**) neutrophils: the control group (saline)—blue, groups with SeNP injection—0.1 mg Se/kg (red), 0.5 mg Se/kg (green), 2.5 mg Se/kg (dark blue). The NETs quantification estimated 0, 7, 14, 21, and 28 days after SeNPs or saline (control) were injected daily. NETs were identified as quantification extracellular DNA of supernatants from neutrophils using fluorometric DNA quantification assay (Pico488 DNA quantification kit) at 503 nm excitation wavelength and at 525 nm detection wavelength. Neutrophils were isolated from eight animals for each experimental group. Data are shown as mean ± SEM. Statistical analysis of experimental groups versus control was performed with paired *t*-test. Significance between group means: *** *p* < 0.001 and * *p* < 0.05.

Another display of the functional activity of the neutrophil is the ability to generate neutrophil extracellular traps (NETs). The DNA concentration contained in NETs released by neutrophils was determined with the Pico488 fluorescence intensity. The amount of extracellular DNA released by unstimulated neutrophils increased during the period of SeNPs being applied at concentrations of 0.1 and 0.5 mg/kg and was significantly higher compared to the control. When animals were injected with a high dose of SeNPs (2.5 mg/kg), a slight change in the ejected NETs was observed; throughout the entire period of SeNP administration, the amount of extracellular DNA slightly increased and remained approximately at the same level from day 7 to day 28 (Figure 6A).

The NET formation during activation in the experimental groups increased dose dependently with the time of SeNP application to mice and was maximal on day 28 for all concentrations (0.1, 0.5, and 2.5 mg/kg SeNPs) compared to the control (Figure 6B). An inversely proportional dependence of the parameter and the SeNP concentration (0.1, 0.5, and 2.5 mg/kg) was registered upon activation of NET release by neutrophils with 1 mg/mL OZ. In particular, the lower the SeNP concentration, the more NET traps were released by cells (Figure 6B). It can be seen that, for a low concentration of SeNPs (0.1 mg/kg), there was a greater increase in the amount of DNA ejected by neutrophils than in the other groups on days 7, 14, 21, and 28 (Figure 6B). A high SeNP concentration (2.5 mg/kg) did not cause significant changes in the NET amount compared to the control; however, it had a tendency to increase, as in other experimental groups (Figure 6B).

**Figure 7:**
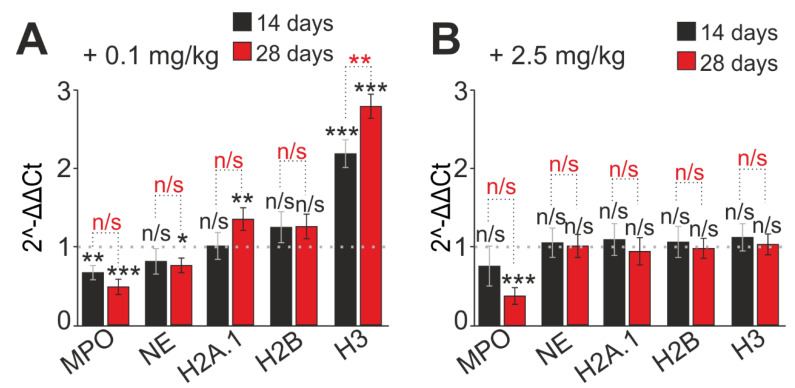
Effect of SeNPs applied in vivo on expression of genes involved in NETs formation. Respective mRNA expression genes encoding H2A.1, H2B, H3, neutrophil elastase (NE), and myeloperoxidase (MPO) in neutrophils 14 (black bars) and 28 (red bars) days after 0.1 mg Se/kg (**A**) and 2.5 mg Se/kg (**B**) SeNPs were injected daily. Dashed line—level of gene expression in controls (mice with administration of saline). GAPDH was used as housekeeping gene. Data are shown as mean ± SEM of five independent experiments. Statistical analysis of experimental groups versus control was performed with paired *t*-test and marked with black asterisks. Significance between experimental groups marked with red asterisks: *** *p* < 0.001, ** *p* < 0.01, and * *p* < 0.05; n/s—no significance.

NETs are web-like extracellular DNA structures coated with histones and enzymes such as neutrophil elastase (NE) and myeloperoxidase (MPO). Therefore, the impact on expression of genes encoding proteins involved in the formation of NET was evaluated. For instance, the respective expression of mRNA of histones H2A.1, H2B, H3, and neutrophilic enzymes MPO and NE changed dynamically during 28 days with the introduction of 0.1 mg/kg SeNPs. A 1.25 and 2.18 times increase in the expression of H2B and H3 histone mRNA was observed on day 14, accordingly, and a 1.26 and 2.78 times increase on day 28 (Figure 7A). Moreover, on day 28, the expression of H2A.1 mRNA also increased by 1.35 times. It was noted that the H3 histone gene turned out to be the most sensitive to the introduction of SeNPs at a low dose. The amount of MPO and NE mRNA was significantly suppressed on both the 14th and 18th days of 0.1 mg/kg SeNPs administration (Figure 7A).

The mRNA level of NET-associated genes changed insignificantly and remained close to the control values after injection of a high dose of SeNPs (2.5 mg/kg) on days 14 and 28 of SeNP administration. The exception was myeloperoxidase, and the expression of the gene encoding it decreased by 24% on the 14th day and by 62% on the 28th day (Figure 7B).

The SeNPs immunostimulatory effect on the neutrophils ability to generate NETs was observed. It was displayed by an increase in their antimicrobial properties, and it correlated with an increase in the expression of histone mRNAs, which are the main NET structure-forming elements.

### 2.5. SeNP Injections into Mice Regulate the Expression of Genes Encoding Selenoproteins

**Figure 8:**
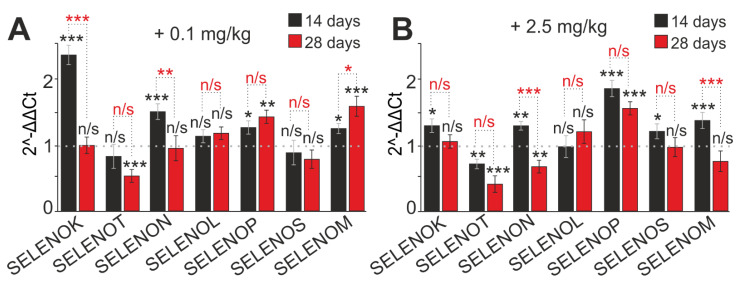
Effect of SeNPs applied in vivo on expression of selenoproteins encoding genes in the neutrophils. Relative mRNA expression of genes encoding selenoproteins 14 (black bars) and 28 (red bars) days after SeNP daily injection in concentrations 0.1 mg Se/kg (**A**) and 2.5 mg Se/kg (**B**). Dashed line—level of gene expression in controls (mice with administration of saline). GAPDH was used as housekeeping gene. Data are expressed as mean ± SEM of five independent experiments. Statistical analysis of experimental groups versus control was performed with paired *t*-test and marked with black asterisks. Significance between experimental groups marked with red asterisks: *** *p* < 0.001, ** *p* < 0.01, and * *p* < 0.05; n/s—no significance.

Selenium and its compounds, entering the body, act as active selenium, which is involved in the synthesis of selenoproteins. Therefore, the expression of mRNA of the selenoproteins that are most important for the neutrophils function was analyzed. Chronic subcutaneous SeNP application for 28 days altered the expression level of neutrophil selenoproteins (Figure 8). Low SeNP concentration (0.1 mg/kg) led to a significant decrease in SELENOТ and SELENIS mRNA on both the 14th and 28th days, whereas the expression of SELENOK, SELENON, SELENOМ, and SELENOP significantly increased on the 14th day, and, in the case of SELENOМ and SELENOP, on day 28 (Figure 8A). The expression of SELENOK and SELENON decreased on the 28th day and became close to the control value. The amount of SELENOL mRNA did not significantly change compared to control cells (Figure 8A).

When animals were injected with a high SeNP concentration (2.5 mg/kg), the expression of the SELENOP gene significantly increased on the 14th and 28th days (by 1.85 and 1.54 times, respectively). On the contrary, the level of SELENOT mRNA decreased (by 29% and 59%, respectively). On the 14th day of 2.5 mg/kg SeNP administration, an increase in SELENOK, SELENON, SELENOS, and SELENOM expression by 1.28, 1.29, 1.19, and 1.36 times, respectively, was observed (Figure 8B). On the 28th day the expression of these genes decreased, and the amount of SELENOK and SELENOS mRNA became close to the control value; in particular, SELENON expression decreased by 32% and SELENOM by 24% (Figure 8B).

Hence, the selenoprotein expression changes depending on the concentration and duration of the SeNP application. This undoubtedly affects the functional state of neutrophils. Selenoprotein P, selenoprotein M, selenoprotein S, selenoprotein K, and selenoprotein T were the most sensitive to the SeNPs introduction into the mouse organism. This highlights that they take part in the maintenance of the functional status of neutrophils. Possibly, they mediate the SeNPs immunomodulatory effect. It is essential to emphasize that the SeNPs immunomodulatory effect on the functional state of neutrophils in vivo depends on their concentration. Moreover, it also depends on the method and duration of administration and is immunostimulatory and antioxidant in nature.

## 3. Discussion

Usually, the method of oral SeNP administration is used in studies on the influence of the source of Se on the functions of immune cells ([18,27,28,48] and many others). On the other hand, injections are also used to avoid loss and settling of highly reactive SeNPs in the digestive tract. It is known that subcutaneous injections are the most preferred method of parenteral drug administration in rodents and rabbits. Subcutaneous administration of substances is characterized by slower absorption than substances administered through other routes such as intravenous or intraperitoneal administration. For subcutaneous injections, small volumes of substances are used, and, as a rule, immunostimulatory compounds are administered in this way. It has been proven that the use of small volumes for subcutaneous administration does not cause physiological disorders in the animal and does not affect zoo-social behavior [49]. It should also be noted that, in most experimental vivariums, rodents are fed with dry complete feed, the use of which requires increased water consumption. When the test solutions are administered subcutaneously, the animal does not irritate the central and peripheral osmoreceptors or experience reflex excitation of the drinking center. In other words, there is no thirst, which usually contributes to experiments with oral administration of drugs [50]. Intravenous and intraperitoneal administrations are often considered equivalent in rodents. Intravenous injections have limitations; they are definitely not suitable for compounds that can cause hemolysis, thrombosis, or vasculitis [49]. It has not been completely defined whether SeNPs are capable of causing such pathological processes directly in the blood; therefore, intravenous injections were not utilized. Important disadvantages during intraperitoneal injection are: a high probability of damage or perforation of internal organs and pain and discomfort in the animal. With prolonged (repeated) use of this method, the drug may accumulate in the abdominal cavity (between the parietal and visceral layers of the peritoneum, on the serous covers of internal organs), abscesses may form, fibrin deposits or drug residues may accumulate on the internal organs, and there is a risk of peritonitis [50]. Two routes of administration of SeNPs, subcutaneous and intraperitoneal, were chosen. The injected solutions were warmed to room temperature to minimize the manifestation of adaptive reactions in the animal. Based on the results, it was concluded that the SeNPs intraperitoneal administration was incorrect for studying the SeNPs immunomodulating effect on neutrophils. This happened due to the fact that this route of SeNP administration led to the development of an inflammatory reaction in the peritoneum, which was confirmed by a significant increase in the number of neutrophils released from the peritoneal cavity (Figure 1A), as well as their activated state without additional actions (Figure 2A). Therefore, most of the experiments were performed on groups of mice with SeNP subcutaneous administration.

### 3.1. Effects of SeNPs and Selenium on the Dynamics of the Adhesive Abilities of Neutrophils

Performing their main function, neutrophils migrate to the site of inflammation along the gradient of chemotactic factors. Leukocyte transmigration is a key process for the infiltration of inflammatory cells into injury sites [13]. Analyzing the results on the number of isolated cells from the abdominal cavity, a trend towards an increase in their number can be discussed. This was especially evident for the administered concentration of 0.1 mg/kg SeNPs (Figure 1). The revealed trend towards an increase in the number of cells can be explained by an increase in the number of neutrophils in the body of mice (or an increase in their lifespan when their apoptosis is suppressed) under the action of SeNPs or an increase in their mobility.

Every day, one billion neutrophils are produced in the bone marrow per 1 kg of body weight. They reach full maturity within 10 days, then they are released from the bone marrow. With the help of constant feedback mechanisms, neutrophils returning to the bone marrow regulate the further formation and death of neutrophils [11,51]. The number of neutrophils can increase to 10 billion during infections, when a large number of inflammatory mediators are produced, including granulocyte-macrophage colony-stimulating factor (G-CSF) [52]. During inflammation, the duration of the circulation of human neutrophils can increase up to 5 days, which can also affect their number in the blood [53]. It has been noted that many genetic mutations, especially in the genes of adhesion molecules, lead to the overproduction of neutrophils [11]. It has been reported that the regulation of the number of neutrophils depends on the number of apoptotic neutrophils, which are phagocytosed by tissue dendritic cells and macrophages. Phagocytosis leads to a decrease in IL23 production, which reduces the production of IL17 by T cells, which leads to a decrease in G-CSF to reduce neutrophil production. A lack of neutrophils entering tissues results in less phagocytosis, increased IL23, IL1, and GCSF and more neutrophil production [54]. It is possible that SeNPs affect the lifespan of neutrophils and change the feedback loop that controls the death and production of neutrophils in the bone marrow or modulate the release of pro-inflammatory cytokines by T lymphocytes. There is a lack of studies demonstrating the relationship between the lifespan of neutrophils and SeNPs. However, the effect of Se on the functional activity of T lymphocytes is widely known and has been described in detail [15,23,55]. It has been shown that Se can affect the migration ability of leukocytes by changing their number at the site of inflammation. For instance, in liver diseases, the recruitment of neutrophils to inflamed tissues increases under the influence of SeNPs. It is assumed that Se is able to increase the intracellular calcium flux of neutrophils, which may enhance the random mobility of neutrophils. The authors of the article considered the possibilities of nanomedical drugs for targeting cell migration and influencing the outcome of liver disease in animal models [13]. Cellular tissue infiltration requires efficient adhesion of circulating blood cells to endothelial cells and subsequent migration to inflammatory sites [26]. The modeling effect of SeNP on neutrophil motility is registered. It has been shown that SeNPs increase the activity of chemotaxis and respiratory burst more significantly than sodium selenite, which indicates a stronger stimulating effect of SeNPs on the functional activity of neutrophils [56]. Furthermore, it has been suggested that the addition of a moderate number of various micronutrients including Se can improve the immunity of dairy cows [12].

The trend towards the increase in the number of neutrophils in the focus of inflammation can be explained by a change in neutrophil adhesive properties, which are also one of the characteristics of the neutrophil functional state. A neutrophil’s adhesive properties are due to the spectrum of adhesion molecules expressed on its surface. All adhesion molecules, including selectins, selectin ligands, and integrins, are thought to be constitutively expressed on neutrophils, binding instantaneously upon neutrophil activation by bacterial products or pro-inflammatory molecules, including histamine, leukotrienes, chemokines, and complement fragments [57]. This allows neutrophils to enter certain areas faster than many other cells, regardless of protein synthesis [11]. The main adhesive molecules of the neutrophil are selectins and integrins. Expression of the adhesion molecules CD62L (L-selectin) and CD11b (b-integrin) is most important for their migration to the inflammation site [58]. Selectins are primarily responsible for the initial phase of the leukocyte adhesion cascade [59]. In turn, CD11b integrin plays a key role in the process of neutrophil transmigration through the endothelium, providing interaction with ICAM1 adhesion molecules located in the endothelium [13,60]. In this study, it is suggested that, if the introduction of SeNPs leads to an increase in their adhesive properties on the 14th day, then SeNPs should modulate (presumably increase) the expression of adhesion proteins, in particular, CD62L and CD11b. Indeed, it was found that both high (2.5 mg/kg) and low (0.1 mg/kg) concentrations of injected SeNPs within 14 days led to a significant increase in the expression of genes encoding adhesive molecules CD62L and CD11b. On the contrary, on day 28, a significant decrease in their expression was observed (Figure 3). It has been shown that long-term Se supplementation may decrease the expression of adhesion molecules. Thus, in a study involving corticoid-dependent asthmatics with low Se circulatory status, Se supplementation (200 μg/day for 6 months) reduced the expression of CD11a, CD11b, and CD62L adhesion molecules on blood mononuclear cells [61]. The authors concluded that Se supplementation may reduce cell migration by suppressing these surface molecules, and this may reflect the mechanism by which increased Se intake (when taken long term) reduces inflammation. During migration to the site of inflammation, neutrophils are usually primed, resulting in a pre-activated and hyper-reactive state [62]. The regulation of the surface expression of molecules characterizing the primed state of the neutrophil depends on the stimuli. For example, in peripheral neutrophils, low doses of platelet-activating factor affect the expression of CD11b but not CD62L. In contrast, low doses of endotoxin affect CD62L but not CD11b [63]. In this particular study, it was observed that low doses of SeNPs administered significantly increased CD62L expression, whereas CD11b was not that significant, and a low dose of SeNPs caused a primed state of neutrophils, which correlated with other results on the assessment of ROS production observed in this research (increased spontaneous production and increased response to stimulus).

It is known that L-selectin genes contain NF-kB binding sites in their promoter regions, which makes them candidates for changing expression in situations accompanied by activation or increase in NF-kB expression, for example, during oxidative stress [59,64]. NF-kB is activated and translocated to the nucleus upon exposure to inflammatory stimuli, one of which is TNF-a [65,66]. In this research paper, it was noted that, on the 14th day of injection of 0.1 or 2.5 mg/kg SeNPs into the animal body, a significant increase in the expression of TNFa and NF-kB mRNA occurred, and, with prolonged administration of SeNPs (28 days), a decrease in the expression of these genes was observed (Figure 7). Thus, there is an unambiguous relationship between adhesion enhancement, an increase in CD62L, TNFa, and NF-kB mRNA on the 14th day, and a decrease in these parameters on the 28th day, which is confirmed by our results. This is in good agreement with the literature data. It has been shown that, in patients with chronic leukemia, there is an increase in the expression of L-selectin on neutrophils, which may be due to a high level of TNF-α in the blood serum [67]. The priming of neutrophils with cytokines leads to increased expression of adhesive molecules, which are involved in interaction with the vascular endothelium and interstitial matrix, on the cytoplasmic membrane, which increases the ability of neutrophils to migrate to the site of inflammation [68,69]. Thus, based on the data received, it is assumed that 0.1 mg/kg SeNPs is a priming dose for neutrophils up to the 14th day of administration; especially CD62L is sensitive to this dose, that is, this dose can modulate the adhesive abilities of neutrophils by increasing their reactivity for short terms of 14 days and decreasing it at long time periods. However, it should be taken into account that, with a longer administration of SeNPs, a significant decrease in the expression of adhesive molecules occurs. Perhaps these results will attract the attention of researchers in relation to the therapy of some diseases where it is necessary to reduce the migration adhesive potential of neutrophils, for example, in cancer or other diseases associated with chronic inflammation.

### 3.2. Effect of SeNPs on ROS Production and Release of Neutrophil Extracellular Traps (NETs)

Neutrophils are specialized cells that generate reactive oxygen species (ROS) at the expense of the NADPH oxidase enzymatic complex. Neutrophil NADPH oxidase is able to produce ROS both spontaneously without any stimulation and through activation of certain receptors [70,71].

A significant dynamic increase in spontaneous and activated ROS production by 1 μM FMLF after administration of all doses of SeNPs for 28 days was observed. This may indicate an immunostimulatory effect of SeNPs on the respiratory burst of neutrophils, which has a cumulative effect. The tripeptide N-formyl-Meth-Leu-Phen (FMLF), derived from bacteria, is a key early chemoattractant that is canonically considered to be of bacterial origin but is also released from damaged mitochondria during tissue necrosis. FMLF initiates an NADPH oxidase-associated respiratory response through the Gi-coupled formyl peptide receptor (FPR1–3) and many signaling components, including protein kinase C (PKC) isoforms and Ca^2+^ ions [2]. Zymosan, a biopolymer isolated from the yeast cell wall, consisting mainly of glucan, is a nonspecific respiratory burst stimulator and activates a repertoire of cell surface receptors (including phagocytic Fc- and complement receptors) [72]. It can be assumed that SeNPs modulate the activity of the respiratory burst of neutrophils and affect the specific signal transduction from FMLF receptors, potentiating it, rather than nonspecific recognition of OZ by neutrophils.

The research published provides quite a wide range of data on the relationship between Se and the ability of neutrophils to generate ROS [56,73,74,75]. For example, in Se-deficient dairy cows, the amount of released ROS and the ability to kill invasive pathogens are significantly reduced, while the addition of Se effectively improves neutrophil chemotaxis and peroxide content [12]. In another study, the timing of exposure is even specified—to create a significant increase in the activity of the respiratory burst of neutrophils with the addition of Se, a period of at least 20 days is required [76], which is in good agreement with our data on the use of SeNPs. There are also papers where the authors compared the effect of sodium selenite and SeNPs on the production of ROS by neutrophils in sheep. Sheep received SeNP (1 mg/kg) or sodium selenite (1 mg/kg) orally for 10 days. It was shown that SeNP increased the activity of chemotaxis and respiratory burst more significantly than sodium selenite, which indicates a stronger stimulating effect of SeNPs on the functional activity of neutrophils [56]. There is evidence of a decrease in ROS production upon exposure to Se. It was shown that, as serum Se concentration increases, ROS production by unstimulated neutrophils decreases [77]. In addition, it has been found that Se can inhibit the NADPH oxidase system. The authors suggested that serum Se not only has the ability to reduce ROS due to antioxidant properties, but also participates in the regulation of the mechanisms of ROS production by neutrophils [78]. The level of ROS production by neutrophils is characterized by their oxidative status, that is, the balance of systems responsible for the production and elimination of ROS. It is known that ROS overproduction can cause oxidative damage to membrane lipids, DNA, proteins, and lipoproteins, leads to oxidative stress, and can cause various diseases [79,80,81]. Effective protection of the body against the toxic effects of ROS is provided by a number of antioxidant systems, including the Se metabolism [82,83,84]. The main enzymes involved in ROS generation are NADPH oxidase (NOX2 and/or NOX4), nitric oxide synthase (NOS), and myeloperoxidase (MPO). The superoxide radical (O2-) is, in most cases, the first ROS produced by NOX. NOS generates NO (nitric oxide), which is an important regulator of neutrophil function and plays a key role in various pathophysiological conditions. NO is produced by three isoforms of NO synthase (NOS1 (neuronal), NOS2 (inducible), and NOS3 (endothelial)), each of which may play different and possibly overlapping roles. However, the distribution, regulation, and functions of NOS in neutrophils are not fully understood. NOS1 and NOS3 are constitutively expressed, while NOS2 is expressed in activated cells and may be elevated in allergic and other inflammatory conditions [85]. In our work, we saw that the expression of NOS2 mRNA was significantly increased on the 14th day, and then, with prolonged injections of SeNP (28 days), it was suppressed. Thus, SeNPs have a stimulating effect on the production of reactive nitrogen species at 14 days, enhancing the antimicrobial potential of neutrophils along with an increase in the expression of NOX2, the main ROS producer in neutrophils. Perhaps this stimulation occurs due to the involvement of selenoproteins in the regulatory protein–protein interactions. With an increase in the time of administration of SeNPs, a redistribution of selenoproteins occurs, since the concentration of Se in the body probably increases, where it manifests its antioxidant properties in a more pronounced form. Interestingly, the expression of NOX2 is suppressed slightly and remains at a higher level than in the control, thus, confirming the immunostimulatory abilities of SeNP in the long term.

Myeloperoxidase (MPO) forms a hypochlorite anion, which, being a strong oxidizing agent, has a nonspecific bactericidal effect. Myeloperoxidase (MPO) is a heme-containing peroxidase expressed mainly in neutrophils and, to a lesser extent, in monocytes. In the presence of hydrogen peroxide and halides, MPO catalyzes the formation of intermediates with active oxygen, including hypochlorous acid (HOCl). The MPO/HOCl system plays an important role in the destruction of microbes by neutrophils. In addition, MPO has been shown to be a local mediator of tissue damage and resulting inflammation in various inflammatory diseases [86]. Its expression was sensitive to SeNP supplementation and was suppressed regardless of the time of administration and concentration; these observations suggest the anti-inflammatory properties of SeNPs.

Evidence of the high anti-inflammatory and antioxidant properties of SeNPs is the increased expression of antioxidant enzymes found by us—SOD2, catalase, HO-1, and Nrf2. It should be noted that the expression of the Nrf2 gene increased significantly in the group with 0.1 mg/kg of SeNPs administered (by 2.9 times), while the effects of enhancing the expression of antioxidant genes were more pronounced than for a concentration of 2.5 mg/kg of SeNPs. This can be explained by the fact that the low concentration of the introduced SeNPs (0.1 mg/kg) also acted here as a priming agent.

Superoxide dismutase (SOD) belongs to the group of antioxidant enzymes. Together with catalase and other antioxidant enzymes, it protects the human body from constantly forming highly toxic oxygen radicals. SOD catalyzes the dismutation of superoxide into oxygen and hydrogen peroxide. Thus, it plays an essential role in the antioxidant protection of almost all cells that are, in one way or another, in contact with oxygen [87]. It is known that SOD2 and HO-1 are induced in response to oxidative stress [88,89]. It is possible that the state of a neutrophil with increased spontaneous activity and increased FMLF-activated ROS production is perceived as being in a state of oxidative stress that acts on the feedback principle—ROS generation triggers activation processes through Nrf-2, expressing antioxidants to suppress the oxidative surge and maintain dynamic redox homeostasis [90]. Modulation of the regulation of NOX expression and activity leads to a change in the level of ROS, which directly or indirectly affects the signaling pathways that determine cell death or survival. Both NOX and Nrf-2 are involved in the regulation of redox homeostasis within cells, and changes in the regulation of their activity can contribute to oxidative stress [87].

The nuclear factor Nrf-2 is an inducible transcription factor that is involved in the transcription of a whole host of antioxidant genes, as it induces the activation of multiple ROS-scavenging pathways that limit oxidative stress and cellular damage. It is also referred to as a redox-sensitive anti-inflammatory transcription factor or an oxidative stress sensor. Se deficiency has been shown to cause neutrophil dysfunction through the suppression of the Nrf2 pathway in pigs [90]. SeNPs, as an antioxidant bioactive compound, have also been shown to increase the activity of reactive-oxygen-species-scavenging enzymes such as glutathione peroxidase, superoxide dismutase, and catalase in the brain of rats and mice [43]. Se itself is an antioxidant that protects cells from reactive oxygen (RO) by reducing free radicals and preventing lipid peroxidation [45].

In 2004, a novel antimicrobial mechanism for neutrophils, the release of neutrophil extracellular traps (NETs), was described. The formation and release of extracellular traps (NETs) characterize the functional activity of the neutrophil [91]. As a rule, the formation of NETs is closely associated with the generation of ROS and the activation of NADPH oxidase and intracellular calcium ion flux [92]. NETs are web-like extracellular DNA structures coated with histones and enzymes such as neutrophil elastase (NE) and myeloperoxidase (MPO) [93].

There is very little information about the effect of Se on NET generation. It has been shown that Se promotes the formation of neutrophil extracellular traps in chicken neutrophils by acting antagonistically towards fumonisin B1. Fumonisin B1 has been shown to inhibit zymosan-induced NET formation in chicken neutrophils, preventing ROS burst and release of histone H3 and neutrophil elastase (NE). The addition of Se reduces the toxic effects of fumonisin B1 and restores NET formation, indicating that Se could be used as a potential drug to prevent fumonisin B1 toxicity in livestock [28].

In another piece of research, the authors studied the effect of trace elements, including Se, on the formation of extracellular traps of neutrophils, which are necessary to maintain their normal vital activity in connection with possible inflammatory diseases such as mastitis, in dairy cows. In this study, low Se concentration (0.01 mg/L) promoted NET formation, while other Se concentrations (0.08 mg/L and 2.0 mg/L) did not affect NET formation compared to PBS. The authors suggested that cow immunity can be improved by adding various micronutrients during cow insemination [29].

It is essential to note the research that investigates the effect of silver nanoparticles (AgNPs) on the induction of neutrophil extracellular traps through the activation of PAD and neutrophil elastase. Silver nanoparticles (AgNPs) are widely used in various fields due to their antimicrobial properties mediated by the formation of ROS. However, many studies have reported that AgNPs can be harmful to both microorganisms and humans. The authors showed that 5 nm AgNPS caused NET release, but 100 nm AgNPs did not [14]. For SeNPs, the dependence of their efficiency on size was also shown [40].

In this particular study a significant increase in spontaneous and activated OD (1 mg/mL) of NET generation was observed at all periods of administration of SeNPs at concentrations of 0.1 and 0.5 mg/kg, while a low concentration of SeNPs (0.1 mg/kg) demonstrated a greater increase in the amount emitted by neutrophils. DNA compared with other groups at all times of SeNP administration. These results also demonstrate the immunostimulatory effect of SeNPs, with 0.1 mg/kg SeNPs acting as a priming agent with the maximum immunostimulatory effect. It should be noted that the stimulated release of NETs in large quantities, presumably, does not harm the surrounding cells, since the main composition of NETs is DNA and H3 and H2A histones but not the NE and MPO enzymes which are responsible for the lytic destruction of surrounding tissues. This assumption is perfectly confirmed by our data on changes in the expression of marker proteins of NET formation under the influence of SeNPs introduced into the body, demonstrating a significant increase in the relative expression of mRNA of histones H2A.1, H2B, and H3 and suppression of the expression of neutrophilic enzymes MPO and NE. A high concentration of administered SeNPs (2.5 mg/kg) tended to manifest anti-inflammatory properties, since the number of NETs practically did not change with time, and nor did the level of expression of NET-associated proteins, but the expression of MPO and NE mRNA was suppressed.

### 3.3. Effect of SeNPs on the Inflammatory Status of Mouse

It is known that ROS levels in neutrophils influence the expression of inflammatory genes. This may occur through the activation of certain transcription factors such as nuclear transcription factor kB (NF-kB) [27]. NF-kB is a pleiotropic regulator of numerous genes involved in immune and inflammatory responses [94,95]. Examining the level of mRNA expression of the factors involved in the regulation of inflammation—TNFa, IL1b, IL6, NF-kB, and SOCS3—under the influence of 0.1 and 2.5 mg/kg SeNPs administered for 14 days, the immunostimulatory effect of SeNPs in the form of an increase in the expression of these factors was noted. However, as is especially important, with long-term administration of SeNPs, we observed an anti-inflammatory effect in the form of suppression of the expression of these factors.

Published data on the expression of inflammatory factors under the action of nanoparticles differ and describe opposite effects. Most likely, the immunomodulating effect of SeNPs depends on the administered dose and duration of administration. On the one hand, it has been shown that stable activation of TNFa expression occurs depending on the dose of nanoparticles and exposure time [96,97]. Another study showed that Se pre-treatment causes a dose-dependent decrease in TNF-α, IL-1β, and sICAM-1 in rat lung injury caused by fine particulate matter [97]. Se can counteract heavy metals and attenuate the inflammatory response. Se also attenuates the cadmium-induced inflammatory response, which is mediated, at least in part, by downregulation of COX-2, iNOS, and TNF-α expression through downregulation of NF-κB activation, suggesting a protective role for Se in the inflammatory process [98,99].

It is suggested that Se supplementation reduces the expression level of key pro-inflammatory genes such as TNFa and cyclooxygenase-2 (COX-2) by blocking the MAP kinase pathways [43]. In addition, in this study, TNF-α was identified as a potent inducer of adhesion molecules, which is consistent with current results; a simultaneous increase in the expression of adhesion molecules and TNFa under the influence of SeNPs (0.1 mg/kg) was noted. Thus, at a low concentration of SeNPs, an immunostimulatory effect of SeNPs was registered, and, at a high concentration (2.5 mg/kg), or with long-term administration, an anti-inflammatory effect was registered. This is in line with the published data; an elevated Se level inhibits NF-kB through glutathione peroxidase, decreases the expression of pro-inflammatory cytokines, and attenuates inflammation [2,43,97].

Most physiological functions of neutrophils depend on their ability to produce and release a range of pro-inflammatory and immunoregulatory cytokines [100]. Even small changes in the synthesis of cytokines by individual neutrophils can lead to a significant modulation of cytokine production given the huge number of neutrophils in the body [101]. This fact confirms the physiological and pathological significance of neutrophils in the pathogenesis of inflammatory, infectious, autoimmune, and neoplastic diseases and makes it possible to identify neutrophils as an important potential target for selective pharmacological intervention aimed at both stimulating and suppressing inflammation. Thus, understanding the modulation mechanisms of cytokines and chemokines by neutrophil-produced SeNPs represents an important step towards a better understanding of how neutrophils can influence pathophysiological processes in vivo.

### 3.4. Effect of SeNP Injections on Selenoprotein Expression in Mouse Neutrophils

Several members of the selenoprotein family regulate or are regulated by cellular Red/Ox tone, which is an important modulator of immune cell signaling and function. There are also important links between selenoproteins and calcium ion (Ca^2+^) flux, which regulates the oxidative release required for optimal immune cell activation [26]. Immune cells express many members of the selenoprotein family; several selenoprotein mRNAs, such as DIO1, 2, and 3, as well as GPX2 and SELV, are not found in immune cells, and some are expressed at very low levels [27]. The main functions of immune cell selenoproteins are antioxidant functions, protein folding, and maintenance of Ca2+ homeostasis [15,26,27,94]. Hugejiletu et al. found that supernutrient Se supplementation alters the expression of selenoproteins involved in innate immunity [27]. Se and its compounds, entering the body, act as active selenium, which is involved in the synthesis of selenoproteins [102,103].

Experiments in this particular study showed that the selenoproteins SELENOT, SELENOM, SELENOP, SELENOS, and SELENOK turned out to be the most sensitive to the effects of SeNPs, which indicates their participation in maintaining the functional status of neutrophils, i.e., it is possible that they provide the immunomodulatory effect of SeNPs. There are only two papers that agree with the discussion of the results of the current study. In chicken neutrophils, GPX2, GPX3, GPX4, Dio1, Dio2, Dio3, Txnrd1, Txnrd2, Txnrd3, SELENOS, SPS2, SELENOK, SEP15, SEPX1, SELENOO, SELENOM, SEPP1, SELENOU, and SELENOH mRNA levels increased, and GPX1 mRNA levels did not change SEPN1, SELENOW1, SELENOT, and SELENOI [104]. Additionally, the addition of supernutritious Se (oral administration of Se–yeast in doses exceeding nutritional doses—>4.9 mg Se/week) altered the expression of selenoproteins in whole blood neutrophils of sheep [27].

The main function of SELENOP is the transport and supply of Se to body cells [105,106]. The transport of SeNPs does not require a specific transporter; however, increased expression of SELENOP may facilitate the spread of Se from the injection site throughout the body. There is increasing evidence that SELENOP not only transports Se, but also performs important antioxidant functions, which are especially important for immune mechanisms and adequate functioning of neutrophils [26,75]. Interestingly, the antioxidant activity of SELENOP plays a crucial role in limiting pathogenicity and oxidative tissue damage during trypanosome infection. Thus, increased expression of SELENOP is observed when mouse macrophages switch from the classical (M1) to the alternatively activated (M2) phenotype. SELENOP has also been shown to affect macrophage migration, possibly by increasing the expression of matrix-associated genes [107]. However, the exact role of SELP in the immune cell population remains rather unclear and requires further study. In the current study, attention was drawn to the significant increase in SELENOP expression during injections of SeNPs for both short and long injection times, and, with an increase in the period of SeNP administration, expression also increased. Perhaps, the cumulative effect of Se during the long-term administration of SeNPs can be discussed. In neutrophils, SELENOP presumably acts as an intracellular antioxidant and adhesion and migration enhancer.

The selenoproteins SELENOK, SELENOS, SELENOT, and SELENOM are endoplasmic reticulum (ER) resident proteins. Despite the similarity of their chemical structure, they have different structural features, different topologies, and perform different functions. It has been repeatedly shown that reduced expression of ER-resident selenoproteins is associated with increased cellular stress and inflammation [15,55]. SELENOM deficiency leads to a decrease in the total antioxidant activity of TXN [108]. It has been shown that human SELENOM over-expression differently regulates the concentration of antioxidants and H2O2, the activity of antioxidant enzymes, and the composition of leukocytes in transgenic rats [109]. Information about the functional role of SELENOM in neutrophils was not found. It can be assumed that SELENOM plays an antioxidant role and, as an ER protein, participates in the processes of proper protein folding and regulation of ER stress.

Another protein, the expression level of which was observed to increase upon exposure to SeNPs, is SELENOK. SELENOK has been shown to be expressed at particularly high levels in mouse immune tissues, suggesting an important role for this selenoprotein in the immune system [110]. SELENOK-deficient macrophages exhibit an impaired oxidative burst during FccR-mediated phagocytosis [111,112,113].

Recent studies have shown that SELENOK is involved in maintaining Ca2+ homeostasis, i.e., promoting Ca^2+^ influx in immune cells upon their activation [110,111,114]. SELENOK is critical for stimulating depot-driven calcium entry (SOCE) into macrophages, T cells, and neutrophils when stimulated by chemokines and when G-linked protein receptor signaling (e.g., the FMLF receptor, as in our case) is triggered in these immune cells. Violation of SOCE in immune cells with SELENOK knockout leads to a decrease in the level of their activation by 50% [111,114]. It is hypothesized that Se can increase the flow of intracellular Ca^2+^ in neutrophils, which enhances the random mobility of neutrophils [113]. Other important cellular functions of SELENOK are participation in the ERAD-associated protein degradation pathway and restoration of the bi-lipid layer of the membrane [111]. Thus, SELENOK can affect Ca^2+^-dependent effector functions of neutrophils, which include ROS generation, mobility and adhesion, and NET release.

SELENOS is also a trans-membrane protein resident in the ER. According to the experiments in this study, a decrease in SELENOS expression was observed. Perhaps it is not as important for the functioning of neutrophils as SELENOK or is less susceptible to the effects of SeNPs. The main function of SELENOS has been defined as removing wrongly folded proteins from the ER lumen, protecting cells from oxidative damage, and participating in ER stress-induced apoptosis [115]. The role of SELENOS in the inflammatory response may be correlated with the phase of inflammation in the wound healing mechanism [116]. Recently, the role of SELENOS in the formation of NETs induced with selenium-deficient arteritis has been shown. Se deficiency leads to a decrease in plasma SELENOS levels, which contributes to the development of cardiovascular diseases due to the activation of neutrophils and increased formation of NET. The authors found that the PPAR pathway is the main mediator of NET formation induced by Se-deficient arteritis [92]. In the current study, a decrease in SELENOS expression was observed against the background of increased mRNA expression of pro-inflammatory cytokines and decreased expression of NF-κB and enhanced NET, which correlates with the published data described above.

SELENOT is an essential oxydoreductase that performs a key Red/Ox function by controlling protein processing in the ER and allowing cells to cope with oxidative stress [117,118]. A decrease in SELENOT expression in transgenic cell and animal models contributes to the accumulation of reactive oxygen and nitrogen species, depletion of calcium reserves, activation of an extensive protein response, and disruption of hormone secretion [119]. Based on the published data, it can be assumed that the reduced expression of SELENOT may contribute to the development of oxidative stress in neutrophils; however, it has been seen that the lack of antioxidant properties of SELENOT is compensated by an increased expression of antioxidant proteins catalase, SOD2, and HO-1. Furthermore, since neutrophils have a short lifespan, and they usually die after they complete their main function, it triggers the apoptosis or netosis processes. Moreover, the reduced expression of SELENOT also contributes to the accumulation of wrongly folded proteins and the development of ER stress processes along the apoptotic pathway.

SELENOI—a selenoprotein responsible for the synthesis of phospholipids—is not an oxidoreductase enzyme but an ethanolamine phosphotransferase involved in the synthesis of two different ethanolamine phospholipids. Until now, SELENOI remains a poorly studied protein. It is unclear how SELENOI might be involved in reprogramming phospholipids to regulate T cell activation and proliferation. The presented data from recent studies show that the activation of T cells leads to an increase in the level of the SELENOI enzyme, i.e., SELENOI has been shown to play an important role in the metabolic reprogramming required for cell proliferation [115,120]. The experiments in the current study showed that SELENOI is expressed in neutrophils at sufficiently high levels, though its expression is practically independent of the introduction of SeNPs. It should be emphasized once again that neutrophils have a huge but not fully explored therapeutic potential. Active targeting of neutrophils can be achieved by modifying their functional status and effect on, for example, neutrophil specific receptors, by involving L-selectin. The exclusive mobility of leukocytes can also be used for another strategy, not only to modify the migration of the neutrophil itself, but also a strategy in which immune cells are used to transport nanoparticles on their own or loaded with a drug to the desired site of action [13]. Currently, neutrophils are not yet the focus of advanced immunotherapy, and additional new basic research may help uncover new neutrophil-based treatment concepts.

## 4. Materials and Methods

All methods were carried out in accordance with relevant guidelines and regulations. Experimental protocols were approved by the Bioethics Committee of the Institute of Cell Biophysics. Experiments were carried out according to Act 708n (23 August 2010) of the Russian Federation National Ministry of Public Health, which states the rules of laboratory practice for the care and use of laboratory animals and the Council Directive 2010/63 EU of the European Parliament on the protection of animals used for scientific purposes.

### 4.1. Experimental Animals

We used 6–7-week-old male mice of outbred strain BALB/c (23–27 g weight). Mice were purchased from the Animals Breeding Center (Branch of Shemyakin and Ovchinnikov Institute of Bioorganic Chemistry, Russian Academy of Sciences, Pushchino, Russia) and were kept in SPF cages measuring 40 × 25 × 15 cm under standard laboratory conditions: a 12 h light circuit, 22 °C. Animals had free access to food and water. All animal care and use protocols were conducted in accordance with the Standards of Humane Care and Use of Laboratory Animals of the Institute of Cell Biophysics, Puschino, Russia. All experiments involving animals also complied with the Guidelines for the Proper Conduct of Animal Experiments of the Bioethics Committee of the Institute of Cell Biophysics and Russian Federation National Ministry of Public Health (Act 708n, 23 August 2010) and the ARRIVE guidelines for reporting animal research.

### 4.2. Preparation and Characterization of Selenium Nanoparticles

Selenium nanoparticles (SeNP) were obtained by laser ablation in deionized water. The solid target was placed at the bottom of a cuvette under a thin layer of water. In this state, the solid target was irradiated with a laser beam (λ = 1064 nm; T = 4–200 ns; f = 20 kHz; P = 20 W; Ep = 1 mJ). The laser beam was mixed on the target using a galvanomechanical scanner TM 2D (Ateko, Moscow, Russia). Depending on the characteristics of the laser radiation and the speed and trajectory of the laser beam, it was possible to obtain colloidal solutions of selenium nanoparticles with specified geometric parameters. The nanoparticle size was characterized using a DC24000 analytical centrifuge (CPS Instruments, Prairieville, LA, USA). The nanoparticle concentration and hydrodynamic radius were evaluated using the Zetasizer Ultra Red Label (Malvern Instruments, Malvern, UK). The morphology of the nanoparticles was studied by electron energy loss spectroscopy using a 200FE transmission electron microscope (Carl Zeiss, Oberkochen, Germany). The nanoparticle size distribution was investigated using a disk analytical centrifuge. The obtained preparation of selenium nanoparticles had a monomodal size distribution. The average nanoparticle size was about 0.1 µm, the half-width was in the range 0.075–0.125 µm. The results obtained were confirmed by dynamic light scattering. The concentration of nanoparticles in the colloid was investigated using a Zetasizer Ultra Red Label; it was shown that, at a concentration of up to 1013 nanoparticles per ml, there was no aggregation in the colloidal solution. Aggregation was not detected during storage of an aqueous colloidal solution of selenium nanoparticles for a month. The zeta potential of the colloidal solution was 16.2 mV. The transmission electron microscope image showed Se nanoparticles with a diameter of about 0.1 µm. Particle sizes shown in the TEM photomicrographs matched the size distribution plotted using an analytical disk centrifuge. The shape of the nanoparticles was spherical. The procedure for obtaining characterization and image of selenium nanoparticles was described in detail earlier [40].

### 4.3. Mouse Model of SeNP Administration

Male mice (weighing 23–27 g) were randomly divided into four groups (each group *n* = 20): the control group, the low dose group (0.1 mg Se/kg), the mid-dose group (0.5 mg Se/kg), and the high-dose group (2.5 mg Se/kg). Every day for 28 days, mice were subcutaneously (s.c.) injected in the area of the withers with saline as a control or SeNPs. To work out the scheme of the experiment and compare the scheme of administration of SeNPs, another experimental group was created (*n* = 20). The mice of this group received SeNPs (2.5 mg Se/kg) injected intraperitoneally (i.p.) also daily for 28 days. On days 7, 14, 21, and 28, five mice from each group were euthanized by decapitation, and their peritoneal neutrophils were isolated.

### 4.4. Isolation of Peritoneal Neutrophils and Estimation of Their Number and Viability

Inflammation in mice of all experimental groups was induced by intraperitoneal injection of zymosan suspension (5 mg/mL, i.p.). After 5 h, the peritoneal cavity was washed with 3 mL Ca2^+^-free Hanks’ balanced salt solution (HBSS, pH 7.4, 4 °C); wash-out was centrifuged at 600 g for 5 min at 4 °C. Purity of PMN population exceeded 95%, as estimated by luminescent microscopy (Leica DM6500, ×40) with anti-Gr-1 antibody (PE-anti-mouse Ly6G/Ly6C) and bisbenzimide H 33258. Survival of cells was 97–99%, as determined by trypan blue staining. The number of neutrophils was estimated by haemocytometer (Reinfeld, Germany) using the trypan blue dye exclusion method. The cell suspension was then separated for the following assays: quantification of adhesion, NETs, ROS production, and total RNA isolation. Isolated cells were kept in HBSS without Ca^2+^ and phenol red for 1 h at 4°C before use.

### 4.5. Adhesion Assay

Samples of neutrophils (3 × 10^5^ cells) suspended in HBSS with 1 mM Ca^2+^ were incubated at 37 °C in a 96-well, flat-bottomed cultural plate (Corning, NY, USA). After incubation for 60 min, supernatant was removed, and 30 µL of ethyl alcohol (96%) was added to each well to fix the neutrophils. Ethyl alcohol was removed after 3 h, and the plate was dried for 12 h at 37 °C. Attached cells were stained with Romanovsky–Himsa dye (1:10 PBS, 100 µL per well) for 40 min, then the dye was removed, and cells were washed three times with PBS. The attached cells were resuspended in 2-propanol (99.5%, 250 L/well), and absorbance of the suspension was measured at 492 nm with the Multiskan Plus spectrophotometer (Labsystem, San-Diego, CA, USA). The absorbance level in the experimental wells was normalized to the same parameter measured in control wells.

### 4.6. Quantification of NETs

NET formation was quantified using Pico488 DNA quantification kit from Lumiprobe RUS Ltd. (Russia & EACU), as described by Alhussien et al. [91] with some modifications. For the ex vivo NET formation study, neutrophils (2 × 10^5^ live cells per well, in triplicate) were seeded in a 96-well, flat-bottomed tissue culture plate (Sigma Aldrich, USA) in RPMI 1640 culture medium containing 2% FBS and lacking phenol red (Thermo Scientific, Waltham, MA, USA). The neutrophils of various experimental groups were incubated for 3.5 h with opsonized zymosan (1 mg/mL; Sigma Aldrich, Burlington, USA) in 100 μL volume at 37 °C and 5% CO2 atmosphere. To disrupt the NETs and facilitate DNA release in the supernatant, 5 U/well of micrococcal nuclease (Sigma Aldrich, Burlington, USA) was added and incubated for 10 min under the same conditions. The samples were then centrifuged (700 *g*, 5 min), and the supernatant (100 μL per well) was transferred into 96-well, flat-bottomed, black polystyrol microplates (Greiner Bio-one, Frickenhausen, Germany). The experimental samples were diluted in 1× TE buffer so that the sample volume was 50% of the measured volume. Then, an equivalent volume of Pico488 dye working solution (100 μL) was added to it, mixed, and incubated in the dark for 5 min. The NET formation was determined in arbitrary fluorescence units (AFU) using an automated multiplate reader (Spark™ 10M multimode microplate reader; Tecan Trading AG, Switzerland, with Spark Control software) at an excitation wavelength of 503 nm and an emission of 525 nm. Unstimulated neutrophils in RPMI medium without phenol red were used as negative controls. We used the DNA Concentration Calculator (https://ru.lumiprobe.com/calc/dna-protein-quantification, accessed on 5 November 2022) to determine the amount of DNA in the NET samples according to the manufacturer’s instructions. Data are expressed as mean of replicate wells ng/mL DNA in supernatant. Data were analyzed from independent experiments using cells isolated from five animals from each experimental group.

### 4.7. Chemiluminescent Analysis of ROS Generation

ROS generation by PMNs was determined by luminol-dependent chemiluminescence, as was described earlier [121]. Spontaneous level induced by 1 µM FMLF (Sigma Aldrich, Burlington, VT, USA) or 1 mg/mL opsonized zymosan (Sigma Aldrich, Burlington, USA) ROS production was studied. In brief, the working mixture contained 169 µL medium with calcium (1 mM), 7 µL luminol (0.35 mM), 2 µL horseradish peroxidase (3 U/mL), 2 µL sodium azide (0.1 mМ), and 20 µL of cells (1 × 10^6^ cells/mL). Neutrophil samples were prepared in triplicate and were distributed in a 96-well, flat-bottomed, white plate (Sigma Aldrich, Burlington, USA). Chemiluminescent analysis of ROS generation by PMNs was estimated by automated multiplate reader (Spark™ 10M multimode microplate reader; Tecan Trading AG, Switzerland, with SparkControl software). After detecting the base level of chemiluminescence intensity, FMLF at concentration of 1 µM or 1 mg/mL opsonized zymosan was added to initiate the respiratory burst. Recording was made continuously during 10–30 min. Each independent experiment was performed with the cells of an individual animal.

Opsonized zymosan was obtained by boiling zymosan powder (10 mg/mL; Sigma Aldrich, USA) suspended in PBS for 20 min, followed by three washes with PBS. Finally, 1 vol mice serum was added to a 10 mg/mL suspension of zymosan in PBS, and the mixture was incubated for 60 min at 37 °C, then washed three times with the incubation buffer and resuspended in the same buffer.

### 4.8. Extraction of RNA and Real-Time Polymerase Chain Reaction (RT-qPCR)

As in our previous works [36], the Mag Jet RNA Kit (Thermo Fisher Scientific, Waltham, MA, USA) was used for the extraction of total RNA from neutrophils. The RNA quality was estimated by electrophoresis in the presence of 1 μg/mL ethidium bromide (2% agarose gel in Tris/borate/EDTA buffer). The concentration of the extracted RNA was determined with NanoDrop 1000c spectrophotometer. RevertAid H Minus First Strand cDNA Synthesis Kit (Thermo Fisher Scientific, Waltham, MA, USA) was used for reverse transcription of total RNA.

Each PCR was performed in a 25 μL mixture composed of 5 μL of qPCRmix—HS SYBR (Evrogen, Moscow, Russia), 1 μL (0.2 μM) of the primer mix, 17 μL water (RNase free), and 1 μL cDNA. Dtlite 5 Real-Time PCR System (DNA-technology, Moscow, Russia) was used for amplification. Amplification process consisted of the initial 5 min denaturation at 95 °C, 40 cycles of 30 s denaturation at 95 °C, 20 s annealing at 60–62 °C, and 20 s extension step at 72 °C. The final extension was performed for 10 min at 72 °C. The sequences of the used primers are presented in Table 1. All the sequences were designed with FAST PCR 5.4 and NCBI Primer-BLAST software. The data were analyzed with Dtlite 5 software (DNA-Technology, Moscow, Russia). The expression of the studied genes was normalized to gene encoding glyceraldehyde 3-phosphate dehydrogenase (GAPDH). Data were analyzed by 2^(-ΔΔCT)^ method, as outlined by Livak and Schmittgen.

### 4.9. Statistical Analysis

All presented data are expressed as mean values ± standard error (SEM) with indicated number of independent experiments carried out on the cells of an individual animal (*n* = 5–8). Statistical analyses were performed by paired *t*-test. MS Excel, ImageJ, Origin 2016 (OriginLab, Northampton, MA, USA), and Prism GraphPad 7 (GraphPad Software, RRID: SCR_002798) software were used for data and statistical analysis.

## 5. Conclusions

For the first time in scientific research, this study demonstrated the immunomodulatory abilities of SeNPs in vivo—an increase in the functional activity of neutrophils at the level of adhesion, ROS production, NET release, and the production of pro-inflammatory factors (cytokines)—and confirmed their antioxidant role in suppressing the expression of mRNA of ROS-generating enzymes and increased expression of antioxidant proteins. The functions of neutrophils can be modulated by SeNPs due to the changes in the spectrum and amount of expression of selenoproteins, which are significantly involved in the control of neutrophil functions. Thus, SeNPs can be considered as potential modulators of neutrophil activity. This is of interest in diseases associated with immunosuppression (chemotherapy in oncology) and inflammation, where neutrophils can be considered as possible therapeutic targets, if their activity is modulated in the desired direction—either activating or suppressing.

## Figures and Tables

**Table 1:** Primer sequences for real-time polymerase chain reaction (RT-PCR).

GAPDH	Forward 5′-tccactcacggcaaattcaac-3′ Reverse 5′-cggcatcgaaggtggaagag-3′
CD62L	Forward 5′-gcagagaactggggtgctgg-3′ Reverse 5′-caactcaggggcctccaaagg-3′
CD11b	Forward 5′-gaagggcatggggctctgaag-3′ Reverse 5′-cagtttgttcccaaaggaggcat-3′
NOX2	Forward 5′-ttgggtcagcactggctctgg-3′ Reverse 5′-caggcccccttcagggttcttg-3′
NOS-2	Forward 5′-gctgcaggtgttcgatgccc-3′ Reverse 5′-ccaaggtagagccatctggctgctt-3′
MPO	Forward 5′-ccttcgtggacgccagcg-3′ Reverse 5′-aagagggtgtgcatggaggtg-3′
HO-1	Forward 5′-ggtcaggtgtccagggaaggct-3′ Reverse 5′-ccctgagaggtcacccaggtagc-3′
Catalase	Forward 5′-gctgacacagttcgtgaccctcg-3′ Reverse 5′- acaggcaagtttttgatgccctggt-3′
SOD2	Forward 5′-ctcccggcacaagcacagc-3′ Reverse 5′-tcctttgggttctccaccaccct -3′
Nrf2	Forward 5′-tcctggacgggactattgaaggctg-3′ Reverse 5′-cacattgggattcacgcataggagcact-3′
TNFa	Forward 5′-acggcatggatctcaaagacaac-3′ Reverse 5′-tcctggtatgagatagcaaatcgg-3′
IL1b	Forward 5′-aatctcgcagcagcacatcaaca-3′ Reverse 5′-tccacgggaaagacacaggtagc-3′
Il6	Forward 5′-aaactctaattcatatcttcaac-3′ Reverse 5′-gtccacaaactgatatgcttag-3′
IL10	Forward 5′-tgtcatcgatttctcccctgtga-3′ Reverse 5′-cattcatggccttgtagacaccttg-3′
NF-kB	Forward 5′-aagtgcaaaggaaacgccagaa-3′ Reverse 5′-actaccgaacatgcctccacca-3′
SOCS3	Forward 5′-aagaacctacgcatccagtgtga-3′ Reverse 5′-atgtagtggtgcaccagcttgag-3′
NE	Forward 5′-catgggctggggcaggttg-3′ Reverse 5′-ggcgaaggcatctgggtacaa-3′
H2A.1	Forward 5′-сcgcaagggcaactactcgg-3′ Reverse 5′-ctcggtcttcttgggcagcag-3′
H2B	Forward 5′-caagtgcaccccgacaccg-3′ Reverse 5′-ctccgacaccgcgtgct-3′
H3	Forward 5′-ccagaagtcgaccgagctgc-3′ Reverse 5′-gatgtccttgggcatgatggtga-3′
SELENOS	Forward 5′-tgggacagcatgcaagaag-3′ Reverse 5′-gcgtccaggtctccagg-3′
SELENOM	Forward 5′-agcctcctgttgcctccgc-3′ Reverse 5′-aggtcagcgtggtccgaag-3′

## Data Availability

The data presented in this study are available on request from the corresponding author.
